# Effects of indole on drug resistance and virulence of *Salmonella enterica* serovar Typhimurium revealed by genome-wide analyses

**DOI:** 10.1186/1757-4749-4-5

**Published:** 2012-05-25

**Authors:** Eiji Nikaido, Etienne Giraud, Sylvie Baucheron, Suguru Yamasaki, Agnès Wiedemann, Kousuke Okamoto, Tatsuya Takagi, Akihito Yamaguchi, Axel Cloeckaert, Kunihiko Nishino

**Affiliations:** 1 Laboratory of Microbiology and Infectious Diseases, Institute of Scientific and Industrial Research, Osaka University, 8-1 Mihogaoka, Ibaraki, Osaka, 567-00447, Japan; 2 Department of Cell Membrane Biology, Institute of Scientific and Industrial Research, Osaka University, Ibaraki, Osaka, Japan; 3 Graduate School of Pharmaceutical Sciences, Osaka University, Suita, Osaka, Japan; 4 INRA, UMR1282 Infectiologie et Santé Publique, F-37380, Nouzilly, France; 5 Université François Rabelais de Tours, UMR1282 Infectiologie et Santé Publique, F-37000, Tours, France

**Keywords:** AcrAB, Indole, RamA, *Salmonella*, SPI-1

## Abstract

**Background:**

Many Gram-positive and Gram-negative bacteria produce large quantities of indole as an intercellular signal in microbial communities. Indole demonstrated to affect gene expression in *Escherichia coli* as an intra-species signaling molecule. In contrast to *E. coli*, *Salmonella* does not produce indole because it does not harbor *tnaA*, which encodes the enzyme responsible for tryptophan metabolism. Our previous study demonstrated that *E. coli*-conditioned medium and indole induce expression of the AcrAB multidrug efflux pump in *Salmonella enterica* serovar Typhimurium for inter-species communication; however, the global effect of indole on genes in *Salmonella* remains unknown.

**Results:**

To understand the complete picture of genes regulated by indole, we performed DNA microarray analysis of genes in the *S. enterica* serovar Typhimurium strain ATCC 14028s affected by indole. Predicted *Salmonella* phenotypes affected by indole based on the microarray data were also examined in this study. Indole induced expression of genes related to efflux-mediated multidrug resistance, including *ramA* and *acrAB*, and repressed those related to host cell invasion encoded in the *Salmonella* pathogenicity island 1, and flagella production. Reduction of invasive activity and motility of *Salmonella* by indole was also observed phenotypically.

**Conclusion:**

Our results suggest that indole is an important signaling molecule for inter-species communication to control drug resistance and virulence of *S. enterica*.

## Background

Bacteria communicate using small molecules by a process termed quorum sensing. Accumulation of quorum-sensing signals in growth medium indicates cell density. The use of chemical signals for bacterial communication is a widespread phenomenon [1-5]. In Gram-negative bacteria, these signals could be *N*-acyl derivatives of homoserine lactone, cyclic dipeptides, and quinolones [6-12]. These signals regulate various functions such as bioluminescence, differentiation, virulence, DNA transfer, and biofilm maturation [13-22].

The intestinal tract is colonized by approximately 10^12^ commensal bacteria including those belonging to the genus *Escherichia*[23-25]. Among *Enterobacteriaceae*, indole is produced by *E. coli* and certain *Proteeae* such as *Proteus vulgarisProvidencia* spp., and *Morganella* spp. [26]. Indole production is commonly used for *Escherichia coli* identification [26]. Indole is generated from tryptophan by the enzyme tryptophanase, encoded by *tnaA*[27]. Extracellular indole is found at high concentrations (over 600 μM) when *E. coli* is grown in enriched medium [28]. Furthermore, indole has also been found in human feces at comparable concentrations (~250–1100 μM) [29,30]. Recent studies have also revealed that indole is an extracellular signal in *E. coli*, since it has been demonstrated to regulate uptake, synthesis, and degradation of amino acids in the stationary phase of planktonic cells [31], multicopy plasmid maintenance, cell division [32], biofilm formation [28], acid resistance [33], and expression of multidrug exporters in *E. coli*[34-36] as well as to regulate the pathogenicity island, including the locus of enterocyte effacement of pathogenic *E. coli*[37,38]. Indole has also been demonstrated as an important cell-signaling molecule for a population-based antibiotic resistance mechanism [39].

*Salmonella enterica* is a bacterial pathogen that causes various diseases in humans including gastroenteritis, bacteremia, and typhoid fever [40]. In contrast to *E. coliS. enterica* does not harbor *tnaA*; therefore, this organism does not produce indole [41]. In our previous study, we demonstrated that an *E. coli*-conditioned medium and indole induced expression of the *acrABtolC* multidrug efflux system of *Salmonella* in a RamA regulator-dependent manner [35]. This suggests that indole is used as a cell-signaling molecule in both intra- and inter-species communication. However, the global effect of indole on *Salmonella* remains to be elucidated.

We hypothesized that indole controls expression of a wide range of genes and plays a role in regulating the physiological functions of *S. enterica* serovar Typhimurium. Therefore, to reveal the complete picture of indole-controlled genes, we conducted microarray analysis of genes affected by indole. Predicted *Salmonella* phenotypes affected by indole based on the microarray data were also examined in this study.

## Methods

### Bacterial strains and growth conditions

*S. enterica* serovar Typhimurium strains used in this study were the wild-type strain ATCC14028s [42] and its derivatives. These included strain NES114 which harbors a FLAG-tag fused at the chromosomal *ramA* gene (*ramA*-FLAG::Km^R^) and strain NES84 which carries a *ramA* reporter plasmid (ATCC 14028s/pNN*ramA*) [35]. Derivatives also included various deletion mutants: *ramA* deletion mutant 14028s∆*ramA*::kan^R^*ramR* deletion mutant 14028s∆*ramR*::kan^R^ and mutant 14028s∆*ram*::kan^R^ deleted of the whole *ram* locus. Bacterial strains were grown at 37 °C in Luria–Bertani (LB) broth supplemented with indole (Sigma) where appropriate.

### DNA microarray analysis

The ATCC 14028s strain was grown in the presence or absence of 1 or 4 mM indole. The cells were rapidly collected for total RNA extraction when the culture reached an optical density (OD) of 0.6 at 600 nm. Total RNA was extracted from the cells using the RNeasy Midi kit (Qiagen) and Turbo DNA-free™ kit (Ambion). After extraction of total RNA, fluorescent labeling of cDNA was performed using the GeneChip DNA labeling reagents (Affimetrix). The fluorescent-labeled cDNA was hybridized in cDNA microarray plates (NimbleExpress™ *S. typhimurium* array; NimbleGen Systems, Inc.). The degree of fluorescence in the plates was measured and quantified using the GeneChip Scanner 3000 (Affymetrix) and GeneChip Operating Software ver. 1.4 (Affymetrix), respectively. Measured values were compared to control values, and p values of distribution of logged data were obtained. A ranked conversion of p values was calculated, and values lying inside 2.5 % of the two extremes were considered valid.

### Semiquantitative RT-PCR

The ATCC 14028s strain was grown in the presence or absence of 2 mM indole. The cells were rapidly collected for total RNA extraction when the culture reached an OD_600_ of 0.6. Total RNA from bacterial cultures was extracted as described above. Semiquantitative RT-PCR was used to measure the transcriptional expression of *rrs* and *ramA*. RNA was reverse-transcribed using random hexamers and TaqMan reverse transcription reagents (Applied Biosystems), and PCR was performed using Takara LA Taq DNA polymerase (Takara Bio, Inc.). The primers for *rrs* were *rrs*-F and *rrs*-R (Table 1) and those for *ramA* were *ramA*-F and *ramA*-R (Table 1).

**Table 1:** Primers used in this study

Primer name	Oligonucleotide sequence (5′ to 3′)
For semiquantitative RT-PCR	
*rrs*-F	CCAGCAGCCGCGGTAAT
*rrs*-R	TTTACGCCCAGTAATTCCGATT
*ramA*-F	ATTTGAATCAGCCGTTACGTATTG
*ramA*-R	TGCAGGTGCCACTTGGAAT
For quantitative RT-PCR	
*gmk*-f	TTGGCAGGGAGGCGTTT
*gmk*-r	GCGCGAAGTGCCGTAGTAAT
*gyrB*-f	TCTCCTCACAGACCAAAGATAAGCT
*gyrB*-r	CGCTCAGCAGTTCGTTCATC
*rrs*-f	CCAGCAGCCGCGGTAAT
*rrs*-r	TTTACGCCCAGTAATTCCGATT
*ramA*-f	GCGTGAACGGAAGCTAAAAC
*ramA*-r	GGCCATGCTTTTCTTTACGA
*acrB*-f	TCGTGTTCCTGGTGATGTACCT
*acrB*-r	AACCGCAATAGTCGGAATCAA
*mdtE*-f	AGTCGCTGGATACCACCATC
*mdtE*-r	GATATTACGCACGCCGATTT
*tolC*-f	GCCCGTGCGCAATATGAT
*tolC*-r	CCGCGTTATCCAGGTTGTTG
*flhC*-f	ATATCCAGTTGGCGATGGAG
*flhC*-r	TTGCTCCCAGGTCATAAACC
*hilA*-f	CATGGCTGGTCAGTTGGAG
*hilA*-r	CGTAATTCATCGCCTAAACG
*invF*-f	TGAAAGCCGACACAATGAAAAT
*invF*-r	GCCTGCTCGCAAAAAAGC
*invA*-f	GGCGCCAAGAGAAAAAGATG
*invA*-r	CAAATATAACGCGCCATTGCT
*sipA*-f	TTTGGCTGTACGTTAGATCCGTTA
*sipA*-r	CCGCCGCTTTGTCAACA

### β-Galactosidase assay

The NES84 strain [35] was grown in the presence of 0–3 mM indole until an OD_600_ of 0.6 was reached. β-Galactosidase activity was determined as described by Miller [43]. All assays were performed in triplicate.

### Construction of the *ramA*-FLAG strain

Insertion of the FLAG-tag of the *ramA* gene 3' terminal was performed as described by Datsenko and Wanner [44]. The kanamycin resistance gene *aph*, flanked by Flp recognition sites, was amplified by PCR using the primers *ramA*-FLAG-forward (GCCAGGCGCTTATCGTAAAGAAAAGCATGGCCGTACGCATGACTACAAGGACGACGATGACAAGTAGGTGTAGGCTGGAGCTGCTTC) and *ramA*-FLAG-reverse (CGATTAAACATTTCAATGCGTACGGCCATGCTTTTCTTTACATATGAATATCCTCCTTAG). The sequence of the FLAG-tag appears in bold in the sequence of the *ramA*-FLAG-forward primer. The resulting PCR products were used to transform the recipient ATCC 14028s strain harboring the pKD46 plasmid, which expresses Red recombinase [44]. The chromosomal structure of the mutated loci was verified by PCR.

### Western blotting

The NES114 strain (*ramA*-FLAG::Km^R^) was grown in the presence of 2 mM indole until an OD_600_ of 0.6 was reached. Bacterial cells were washed with buffer [20 mM Tris–HCl (pH 8.0), 200 mM NaCl, and 1 mM EDTA], resuspended in 1 ml of the same buffer, and disrupted by sonication using the Branson Sonifier 200 (Branson Sonic Power Co., Danbury, CT, USA) on ice for 2.5 min. Whole-cell lysate (10 μg of protein) was separated on 15 % SDS-PAGE using Tris-glycine SDS as the running buffer. The gel was transferred to PVDF membranes, and analyzed by western blotting using a monoclonal anti-FLAG antibody (Sigma). The blot was developed using anti-mouse IgG horseradish peroxidase-conjugated antibody and analyzed using the ECL detection system (GE Healthcare).

### Transmission electron microscopy

One hundred microliters of an overnight culture of the wild-type *Salmonella* strain was added to 5 ml of LB broth, and the bacterial culture was grown in the presence or absence of 1 mM indole until an OD_600_ of 0.6 was reached. Bacterial cells were collected by centrifugation, fixed in 2 % glutaraldehyde solution, and observed using a transmission electron microscope JEM-2100 (JEOL Ltd.).

### Gene expression analysis by qRT-PCR

Bacteria were grown until mid-log phase (OD_600_ of 0.6) and harvested by centrifugation. Pelleted cultures were stabilized with RNAprotect Bacteria Reagent (Qiagen) and stored at −80 °C until use. Total RNA was extracted using the RNeasy Mini kit. (Qiagen) following the manufacturer’s instructions. Removal of residual genomic DNA was performed using the Turbo DNA-free kit (Ambion) and examined by negative PCR amplification of a chromosomal sequence. RNA integrity was examined by electrophoresis on 1 % agarose gel. Total RNA was reverse-transcribed using random hexamers and the Superscript III First Strand Synthesis System (Applied Biosystems). Primers used for qRT-PCR are listed in Table 1. The cycling conditions were as follows: 95 °C for 5 min followed by 40 cycles of 95 °C for 10 s and 60 °C for 15 s. After each run, amplification specificity and the absence of primer dimers were examined using a dissociation curve acquired by heating the PCR products from 60 to 95 °C. The relative quantities of transcripts were determined using the standard curve method and normalized against the geometric mean of three reference genes (*gmk*, *gyrB*, *rrs*). In qRT-PCR experiments performed to address the effect of 1 mM indole, the expression level of each gene of interest was calculated as the average of three independent RNA samples. A two-tailed Student’s *t*-test was used to assess significance using a *p* value of <0.05 as a cutoff.

### Measurement of motility of *Salmonella*

An overnight culture of the ATCC 14028s *Salmonella* strain was diluted in LB broth and grown in the presence or absence of 1 mM indole until an OD_600_ of 0.6 was reached. Next, 1 μl of bacterial culture was spotted in the center of a semi-solid agar plate containing 1 % tryptone peptone and 0.3 % BactoAgar and incubated at 37 °C for 3–5 h in a humidified incubator, after which strains were assessed for motility.

### Invasion assay

Invasions assays were essentially performed as previously described [45]. Caco-2 cells were grown in Dulbecco’s modified Eagle medium (DMEM) supplemented with 10 % inactivated fetal bovine serum, 1 % nonessential amino acids, and 1 % antibiotic solution (Gibco, Invitrogen). Cells harvested by trypsinization were seeded at 2 × 10^5^ cells/well in a 24-well plate (Falcon) and incubated for 4 days at 37 °C under 5 % CO_2_ in the medium described above, to obtain a confluent monolayer. Antibiotic was removed 24 h before performing the invasion assays. Bacteria were grown to an OD_600_ of 0.6 in LB broth in the presence or absence of 1 mM indole. After washing with DMEM, bacteria were inoculated on Caco-2 cells at a multiplicity of infection of 30, and the plates were further incubated for 30 min. The bacteria-containing medium was removed from the wells, and the cells were washed with PBS. Cells were incubated for 1.5 h with DMEM supplemented with 100 μg/ml gentamicin. Cells were washed with PBS and lysed by the addition of sterile ultrapure water for 30 min. Serial dilutions were plated on LB agar. The percentage of penetrating bacteria was calculated on the basis of the ratio of the counted cfu to the bacterial inoculum. For each bacterial strain and for each condition, three replicates were used.

## Results

### Indole affects gene expression in *Salmonella*

The regulation of *Salmonella* genes in the wild-type strain ATCC 14028s by 1 or 4 mM indole, was analyzed using DNA microarray. To exclude noise data based on microarray analysis, we considered that genes for which expression changed in the presence of both 1 and 4 mM of indole were significantly regulated by indole. As a result, it was revealed that 24 (Table 2) and 53 genes (Table 3) were upregulated and downregulated by indole, respectively.

**Table 2:** ***Salmonella*genes whose relative expression was increased by indole**

STM no.	Gene	Function	Effect of indole on gene expression (fold change)	
			Concentration of indole (mM)	
			1	4
STM0521	*ybbV*	Putative cytoplasmic protein	8.6	7.0
STM0581	*ramA*	Transcriptional regulator (activator) of *acrAB* and *tolC* (AraC/XylS family)	7.0	39
STM0584	*entD*	Enterochelin synthetase, component D (phoshpantetheinyltransferase)	6.1	7.5
STM0707	*kdpF*	Putative outer membrane protein	8.6	18
STM0823	*ybiJ*	Putative periplasmic protein	3.2	11
STM1156	*yceA*	Putative enzyme related to sulfurtransferases	4.3	8.0
STM1214	*ycfR*	Putative outer membrane protein	4.6	37
STM1251		Putative molecular chaperone (small heat shock protein)	11	9.2
STM1355	*ydiP*	Putative transcription regulator, AraC family	11	7.5
STM1472		Putative periplasmic protein	7.5	15
STM1790	*hyaE*	Putative thiol-disulfide isomerase and thioredoxins	7.0	9.2
STM1868A		Putative protein	4.3	7.0
STM2103	*wcaJ*	Putative UDP-glucose lipid carrier transferase/glucose-1-phosphate transferase in colanic acid gene cluster	4.0	5.3
STM2106	*wcaI*	Putative glycosyl transferase in colanic acid biosynthesis	4.6	34
STM2206	*fruF*	Phosphoenolpyruvate-dependent sugar phosphotransferase system, EIIA 2	4.6	12
STM3028	*stdB*	Putative outer membrane usher protein	6.5	6.5
STM3444	*bfd*	Regulatory or redox component complexing with Bfr, in iron storage and mobility	8.0	20
STM3511	*yhgI*	Putative thioredoxin-like proteins and domain	5.7	8.0
STM3606	*yhjB*	Putative transcriptional regulator (LuxR/UhpA familiy)	7.0	20
STM3668	*yiaK*	Putative malate dehydrogenase	7.0	9.2
STM3941		Putative inner membrane protein	8.6	18
STM4213		Putative phage tail sheath protein	6.5	5.7
STM4327	*fxsA*	Suppresses F exclusion of bacteriophage T7	4.9	5.7
STM4548	*bglJ*	Transcriptional regulator (activator) of *bgl* operon (LuxR/UhpA family)	26	5.7

**Table 3:** ***Salmonella*genes whose relative expression was decreased by indole**

STM no.	Gene	Function	Effect of indole on gene expression (fold change)	
			Concentration of indole (mM)	
			1	4
STM0701	*speF*	Ornithine decarboxylase isozyme, inducible	0.35	0.038
STM0964	*dmsA*	Anaerobic dimethyl sulfoxide reductase, subunit A	0.12	0.063
STM0965	*dmsB*	Anaerobic dimethyl sulfoxide reductase, subunit B	0.13	0.025
STM1092	*orfX*	Putative cytoplasmic protein	0.082	0.031
STM1171	*flgN*	Flagellar biosynthesis: belived to be export chaperone for FlgK and FlgL	0.27	0.082
STM1183	*flgK*	Flagellar biosynthesis, hook-filament junction protein 1	0.19	0.031
STM1184	*flgL*	Flagellar biosynthesis; hook-filament junction protein	0.18	0.044
STM1626	*trg*	Methyl-accepting chemotaxis protein III, ribose and galactose sensor receptor	0.15	0.054
STM1732	*ompW*	Outer membrane protein W; colicin S4 receptor; putative transporter	0.29	0.047
STM1764	*narG*	Nitrate reductase 1, alpha subunit	0.095	0.041
STM1765	*narK*	MFS superfamily, nitrite extrusion protein	0.058	0.047
STM1917	*cheB*	Methyl esterase, response regulator for chemotaxis (cheA sensor)	0.27	0.067
STM1918	*cheR*	Glutamate methyltransferase, response regulator for chemotaxis	0.14	0.0078
STM1919	*cheM*	Methyl accepting chemotaxis protein II, aspartate sensor-receptor	0.18	0.018
STM1921	*cheA*	Sensory histitine protein kinase, transduces signal between chemo- signal receptors and CheB and CheY	0.18	0.029
STM1922	*motB*	Enables flagellar motor rotation, linking torque machinery to cell wall	0.15	0.021
STM1923	*motA*	Proton conductor component of motor, torque generator	0.20	0.036
STM1960	*fliD*	Flagellar biosynthesis; filament capping protein; enables filament assembly	0.31	0.011
STM1961	*fliS*	Flagellar biosynthesis; repressor of class 3a and 3b operons (RflA activity)	0.31	0.019
STM1962	*fliT*	Flagellar biosynthesis; possible export chaperone for FliD	0.25	0.038
STM2256	*napB*	Periplasmic nitrate reductase, small subunit, cytochrome C550, in complex with NapA	0.23	0.082
STM2257	*napH*	Ferredoxin-type protein: electron transfer	0.12	0.067
STM2258	*napG*	Ferredoxin-type protein: electron transfer	0.077	0.027
STM2259	*napA*	Periplasmic nitrate reductase, large subunit, in complex with NapB	0.072	0.033
STM2260	*napD*	Periplasmic nitrate reductase	0.063	0.0078
STM2261	*napF*	Ferredoxin-type protein: electron transfer	0.044	0.024
STM2872	*prgJ*	Cell invasion protein; cytoplasmic	0.22	0.10
STM2873	*prgI*	Cell invasion protein; cytoplasmic	0.18	0.041
STM2874	*prgH*	Cell invasion protein	0.082	0.0078
STM2885	*sipB*	Cell invasion protein	0.33	0.11
STM2897	*invE*	Invasion protein	0.25	0.038
STM2899	*invF*	Invasion protein	0.12	0.095
STM3127		Putative cytoplasmic protein	0.29	0.058
STM3128		Putative oxidoreductase	0.14	0.036
STM3129		Putative NAD-dependent aldehyde dehydrogenase	0.14	0.047
STM3149	*hybA*	Function unknown, intitally thought to be hydrogenase-2 small subunit which now identified as hybO	0.25	0.025
STM3216		Putative methyl-accepting chemotaxis protein	0.16	0.038
STM3217	*aer*	Aerotaxis sensor receptor, senses cellular redox state or proton motive force	0.13	0.047
STM3242	*tdcD*	Propionate kinase/acetate kinase II, anaerobic	0.14	0.029
STM3243	*tdcC*	HAAAP family, L-threonine/L-serine permease, anaerobically inducible	0.082	0.044
STM3244	*tdcB*	Threonine dehydratase, catabolic	0.063	0.029
STM3245	*tdcA*	Transcriptional activator of tdc operon (LysR family)	0.18	0.095
STM3577	*tcp*	Methyl-accepting transmembrane citrate/phenol chemoreceptor	0.13	0.041
STM3626	*dppF*	ABC superfamily (atp_bind), dipeptide transport protein	0.31	0.036
STM3628	*dppC*	ABC superfamily (membrane), dipeptide transport protein 2	0.33	0.088
STM4258		Putative methyl-accepting chemotaxis protein	0.08	0.088
STM4300	*fumB*	Fumarase B (fumarate hydratase class I), anaerobic isozyme	0.19	0.088
STM4305		Putative anaerobic dimethyl sulfoxide reductase, subunit A	0.14	0.082
STM4306		Putative anaerobic dimethyl sulfoxide reductase, subunit B	0.11	0.058
STM4452	*nrdD*	Anaerobic ribonucleoside-triphosphate reductase	0.047	0.095
STM4465		Putative ornithine carbamoyltransferase	0.19	0.10
STM4466		Putative carbamate kinase	0.19	0.10
STM4467		Putative arginine deiminase	0.082	0.019

### Genes upregulated by indole

Expression of the 24 genes listed in Table 2 was upregulated by both 1 and 4 mM indole. Eighteen genes are considered to code for putative proteins, and four genes, *ramAydiPyhjB*, and *bglJ*, are regulatory in nature. The transcriptional activator RamA, which belongs to the AraC/XylS family of regulatory proteins, promotes multidrug resistance by increasing expression of the AcrAB–TolC multidrug efflux system in several pathogenic *Enterobacteriaceae*[35,46-53]. RamA has also been reported to negatively affect virulence [47]. Both YdiP and YhjB are putative transcription regulators that belong to the AraC and LuxR/UhpA families, respectively (Table 2). YhjB is considered a response regulator comprising a CheY-like receiver domain and a helix-turn-helix DNA-binding domain. YhjB in *E. coli* stimulates dephosphorylation of two histidine kinases, EnvZ and NtrB, although the sensor kinase for YhjB phosphorylation has not yet been identified [54]. BglJ forms a heterodimer with RcsB to relieve repression of the *E. coli bgl* operon and allow arbutin and salicin transport and utilization [55,56]. Expression of all these regulatory genes increased by more than 10-fold in the presence of indole. Of particular interest, expression of *ramA* increased by 39-fold in response to 4 mM indole. Noticeably, higher indole concentration did not always lead to a higher expression level. Indeed, *bglJ* expression was more increased in response to 1 mM indole (26-fold) than to 4 mM indole (5.7-fold).

Among other genes upregulated by indole, functions of the gene products of *entDfruFbfd*, and *fxsA* have been characterized. EntD has phosphopantetheinyl transferase activity [57] and is involved in biosynthesis of the iron-acquiring siderophore enterobactin [58]. FruF is a bifunctional PTS system fructose-specific transporter subunit IIA/HPr protein [41,59]. Bfd is a bacterioferritin-associated ferredoxin considered to be involved in Bfr iron storage and release functions or in regulation of Bfr [41]. FxsA of *E. coli* was described as a suppressor of the F exclusion of phage T7 [60]. Expression of all these genes was more strongly induced by 4 mM indole than by 1 mM indole.

### Genes downregulated by indole

Microarray analysis revealed that fifty-three genes were repressed by both 1 and 4 mM indole (Table 3). Expression of all these genes excluding STM4258 and *nrdD* was reduced by 4 mM indole compared to that by 1 mM indole. Although there are 10 putative genes, functions of most gene products have been characterized (Table 3).

Microarray analysis revealed that indole represses expression of genes related to bacterial motility including flagella biosynthesis (*flgN/K/L* and *fliD/S/T*), chemotaxis (*cheB/R/M/Aaer*[61], *tcp*, and *trg*), and flagella motor activity (*motB/A*). Indole also decreased expression of genes related to cell invasion such as *prgJ/I/HsipB*, and *invE/F*, which are encoded by the *Salmonella* pathogenicity island 1 (SPI-1). Expression of genes related to anaerobic respiration was decreased by indole. The genes repressed by indole included *narG* (nitrate reductase), *narK* (nitrate-nitrite antiporter), and genes in the *nap* operon encoding nitrate reductase (Nap) such as *napB/H/G/A/D/F*[62]. The *tdc* operon including *tdcA/B/C/D*, which are responsible for the anaerobic degradation of threonine [63], was also downregulated by indole. In addition to these genes, other genes related to anaerobic respiration such as *dmsA/B* (anaerobic dimethyl sulfoxide reductase), *fumB* (fumarase B, anaerobic isozyme), *nrdD* (anaerobic ribonucleoside-triphosphate reductase), and STM4305/4306 (putative anaerobic dimethyl sulfoxide reductase, subunit A/B) were also repressed by indole. Indole also repressed membrane protein genes such as *ompW* (outer membrane protein involved in osmoregulation that is also affected by environmental conditions) and *dppF/C* (dipeptide transport protein).

### Indole upregulates genes involved in efflux-mediated multidrug resistance

**Figure 1:**
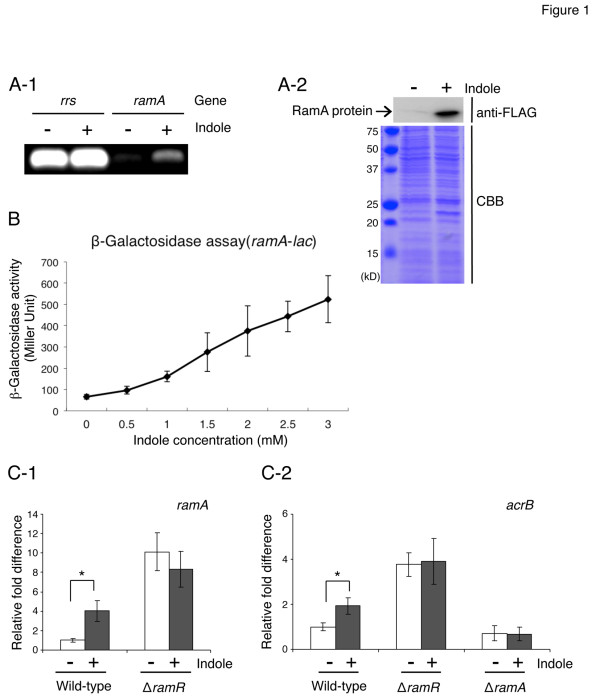
**Indole induces efflux-mediated multidrug resistance genes.** (**A-1**) RT-PCR measurement of indole effect on expression of *ramA*. Expression of *rrs*, encoding rRNA, was measured as a control. The wild-type strain ATCC14028s was grown in the presence (+) or absence (−) of 2 mM indole, and RT-PCR was performed after RNA isolation. (**A-2**) RamA production in the wild-type ATCC14028s derivative strain carrying the epitope-tagged *ramA*. NES114 (*ramA*-FLAG::Km^R^) was grown in the presence (+) or absence (−) of 2 mM indole. SDS-PAGE of lysates of NES114 was followed by Western blotting with an anti-FLAG antibody (anti-FLAG, top) or by staining with Coomassie brilliant blue (CBB, bottom). (**B**) β-Galactosidase levels in the wild-type ATCC 14028s derivative strain carrying the *ramA*-*lac* transcriptional fusion (NES84) treated with different indole concentrations. (**C-1**) qRT-PCR measurement of indole effect on expression of *ramA*. The wild-type strain (ATCC14028s) and its *ramR::kan*^*R*^ deletion mutant were grown in the presence (+) or absence (−) of 1 mM indole. (**C-2**) qRT-PCR measurement of indole effect on expression of *acrB*. The wild-type strain ATCC14028s and its *ramR::kan*^*R*^ and *ramA::kan*^*R*^ deletion mutants were grown in the presence (+) or absence (−) of 1 mM indole. (**B** and **C-1, 2**) The data correspond to mean values from three independent replicates. The bars indicate the standard deviation. (**C-1, 2**) *ramA* and *acrB* expression levels were expressed relative to that measured in the wild-type strain grown without indole, which was assigned the unit value. Asterisks indicate statistically significant difference (*p* < 0.05) according to a two-tailed Student’s *t*-test.

**Figure 2:**
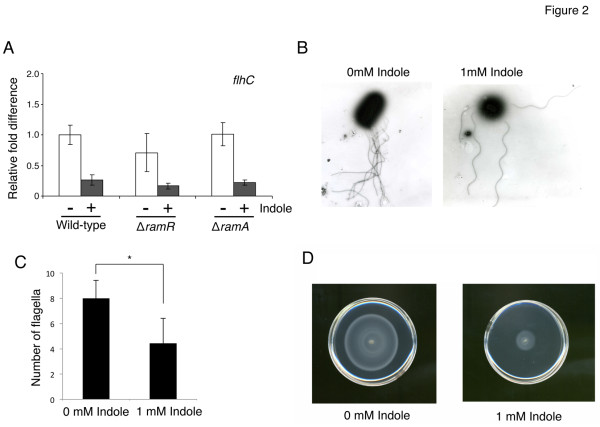
**Indole represses flagella production and motility of*Salmonella*.** (**A**) The effect of indole on expression of *flhC* measured by qRT-PCR. The wild-type strain ATCC 14028s and its *ramR::kan*^*R*^ and *ramA::kan*^*R*^ deletion mutants were grown in the presence (+) or absence (−) of 1 mM indole. *flhC* expression level was expressed relative to that measured in the wild-type strain grown without indole, which was assigned the unit value. The data corresponds to mean values from three independent replicates. The bars indicate the standard deviation. (**B**) Transmission electron microscopy was used to detect flagella on the wild-type strain (ATCC 14028s) grown in the presence or absence of 1 mM indole. (**C**) The number of flagella attached to a single cell was counted from images taken using transmission electron microscopy. Data were collected from 30 bacterial cells for both indole-treated and untreated cells. Bars correspond to the standard deviation. Asterisks indicate statistically significant differences (*p* < 0.01) according to the two-tailed Student’s *t*-test. (**D**) Indole represses motility of *Salmonella*. After incubation of the wild-type ATCC 14028s strain in the presence or absence of 1 mM indole, motility was assayed on a semi-solid agar plate. Result is representative of one of the three experiments.

As reported above, microarray analysis identified that indole significantly increased expression of *ramA*, encoding a transcriptional activator of the multidrug transporter genes *acrAB* and *tolC* of *Salmonella*[35]. To confirm this result, we performed reverse transcriptase polymerase chain reaction (RT-PCR) and observed that transcript levels of *ramA* increased when bacterial cells of the strain ATCC 14028s were treated with 2 mM indole (Figure 1A-1). To investigate whether indole induces production of RamA, we constructed a strain NES114 that harbors a FLAG-tag fused to chromosomally-encoded *ramA*. Western blotting revealed increased production of RamA in the presence of 2 mM indole (Figure 1A-2). Microarray analysis demonstrated that expression of *ramA* was more strongly induced by 4 mM indole (39-fold increase relative to untreated cells) than by 1 mM indole (7.0-fold increase), indicating that the effect of indole on expression of *ramA* may be concentration dependent. To investigate the effect of different indole concentrations on the promoter activity of *ramA*, a β-galactosidase assay with the NES84 strain [35] was performed, and it was found that indole activated the *ramA* promoter in a concentration-dependent manner (Figure 1B). The finding of enhanced promoter activity of *ramA* is in good agreement with our previous observation [35].

Confirming classical RT-PCR results, quantitative RT-PCR (qRT-PCR) indicated that expression of *ramA* in the strain ATCC 14028s increases by 4-fold in the presence of 1 mM indole (Figure 1C-1). As previously reported, RamR represses to the same extent the expression of *ramA*[46,64]. Therefore, to determine a possible contribution of RamR to induction of expression of *ramA* via indole, the effect of 1 mM indole on expression of *ramA* in a ∆*ramR* strain (14028s∆*ramR*::kan^R^) was examined. At this concentration indole did not induce expression of *ramA* suggesting indole-mediated *ramA* induction is indeed dependent on the presence of RamR (Figure 1C-1). However, whereas indole was shown to induce expression of *acrB* in the wild-type strain, this induction was neither observed in the ∆*ramR* (14028s∆*ramR*::kan^R^) nor in the ∆*ramA* strain (14028s∆*ramA*::kan^R^) (Figure 1C-2). This suggested that indole-mediated induction of *acrB* expression is not solely dependent on RamA, but nevertheless also requires the presence of the RamR transcriptional repressor.

### Indole represses motility of *Salmonella*

Expression of genes related to bacterial flagella biosynthesis, flagella motor activity, and chemotaxis was repressed by indole, and this repression was predicted to have profound negative effects on flagellar synthesis and bacterial motility. FlhC is a master regulator protein involved in flagellar biogenesis in *Salmonella*[65]. Indole also reduced expression of *flhC*, and this reduction was independent of *ramA* and *ramR* (Figure 2A). To confirm the microarray findings, we examined the effect of indole on the presence of flagella in wild-type *Salmonella* cells by transmission electron microscopy (Figure 2B). Flagella were detectable in bacterial cells regardless of indole treatment; however, the number of flagella decreased when cells were treated with indole (Figure 2B and C). It was observed that motility of *Salmonella* ATCC 14028s strain decreased in the semi-solid agar plate when bacterial cells were treated with indole (Figure 2D). These results suggest that the reduction in the number of flagella by indole may affect motility of *Salmonella*.

### Indole represses expression of the invasion genes

**Figure 3:**
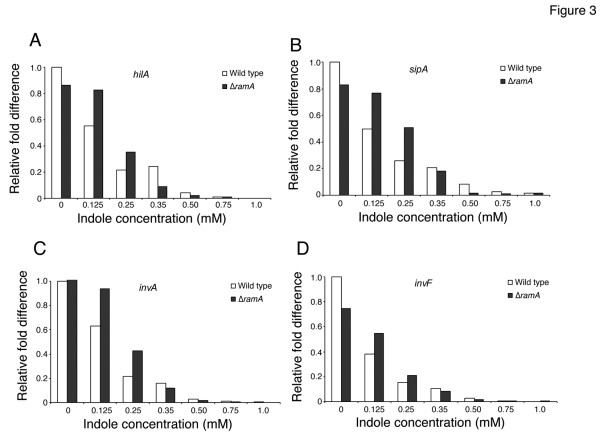
**Indole represses expression of invasion genes encoded by SPI-1.** The effect of indole on expression of SPI-1 genes including *hilA* (**A**), *sipA* (**B**), *invA* (**C**), and *invF* (**D**) was measured by qRT-PCR. The wild-type ATCC 14028s strain (open bars) and its *ramA::kan*^*R*^ deletion mutant (solid bars) were grown with indole concentrations between 0 and 1 mM. Genes expression levels were expressed relative to that measured in the wild-type strain grown without indole, which was assigned the unit value.

Inside the host, *S. enterica* serovars can invade and survive in epithelial cells and macrophages. Therefore, invasion of the host intestinal cells is critical for initiation of salmonellosis. Several genetic elements responsible for the invasive phenotype of *S. enterica* serovar Typhimurium are located in SPI-1, a 40-kbp region of the chromosome at centrisome 63. As described above, we found that genes located in SPI-1 such as *prgJ/I/HsipB*, and *invE/F* were repressed by indole. To further investigate repression of SPI-1 genes by indole, we measured expression of *hilAsipAinvA*, and *invF* of the ATCC 14028s strain in response to different indole concentration by qRT-PCR (Figure 3). *hilA* is located on SPI-1, and it encodes the HilA regulator, which controls expression of SPI-1 genes, including the type III secretion system (T3SS). *invF* also encodes an invasion regulatory protein of SPI-1. *sipA* encodes an effector protein, the secretion of which is mediated by T3SS. *invA* encodes a structural component of T3SS. qRT-PCR revealed that indole decreased expression of *hilAsipAinvA*, and *invF* in a concentration-dependent manner (Figure 3). Since it was reported that overexpression of *ramA* results in decreased expression of SPI-1 genes [47] and our study indicated that indole induces *ramA*, we investigated the effect of *ramA* deletion on expression of SPI-1 genes regulated by indole. As shown in Figure 3, indole repressed expression of *hilAsipAinvA*, and *invF*; however, its repressive effect on those in the *ramA*-deleted mutant (14028s∆*ramA*::kan^R^) was slightly lower than that observed in the wild-type strain (ATCC 14028s) when bacterial cells were treated with 0.125 or 0.25 mM indole. In contrast, when bacterial cells were treated with concentrations of indole of 0.35 mM or more, the repressive effect on SPI-1 genes was similar in the wild-type and in the mutant strains. These data suggest that indole partially represses SPI-1 genes in a RamA-dependent manner when cells are treated with lower indole concentrations; however, the repressive effect of indole on SPI-1 may be RamA-independent at higher concentrations.

**Figure 4:**
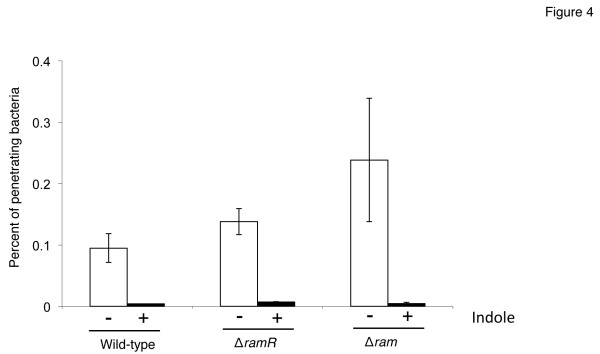
**Indole reduces invasive activity of*Salmonella*cells.** In vitro invasion of Caco-2 cells by the wild-type ATCC 14028s strain and its *ramR::kan*^*R*^ and *ram::kan*^*R*^ deletion mutants grown in the presence (+, solid bars) or absence (−, open bars) of 1 mM indole. The percentage of intracellular bacteria was determined after infecting Caco-2 human intestinal epithelial cells and gentamicin treatment. Results are representative of a single experiment where each strain was tested in triplicates.

A critical step in *Salmonella* pathogenesis is invasion of enterocytes and M cells of the small intestine via expression of a type III secretion system encoded by SPI-1 that secretes effector proteins into host cells, leading to engulfment of bacteria within large membrane ruffles. As indicated previously, indole represses expression of genes encoded by SPI-1, suggesting that indole reduces invasion of mammalian cells by *Salmonella*. To examine this possibility, we investigated the effect of indole in an invasion assay using Caco-2 cells. When bacterial cells were treated with 1 mM indole, the invasion rate of *Salmonella* was reduced compared to that in untreated bacterial cells (Figure 4). We also examined the effect of deletions of *ramR* and of the whole *ram* locus on invasive activity of *Salmonella* treated with 1 mM indole. Indole repressed invasive activity of the ∆*ramR* (14028s∆*ramR*::kan^R^) and of the ∆*ram* mutant (14028s∆*ram*::kan^R^), as observed with the wild-type strain (Figure 4). These data suggest that 1 mM indole phenotypically represses invasive activity of *Salmonella* in a *ram* locus-independent manner.

## Discussion

Increasing evidence indicates that indole controls various phenotypes of *E. coli* including multidrug resistance and virulence as an extracellular signal [28,31-34,37-39]. In contrast to *E. coli*, the effect of indole on *Salmonella* had not been clearly elucidated, probably because *Salmonella* does not produce indole. Therefore, we sought to resolve the effect of indole on gene expression in the *S. enterica* serovar Typhimurium ATCC 14028s strain by microarray analysis. As hypothesized and confirmed in previous studies [35,36], microarray analysis revealed that indole increased expression of *ramA*, which is involved in regulation of the AcrAB–TolC multidrug efflux system.

Including *ramA*, 24 genes were upregulated by indole. Among them, 18 were more strongly induced by 4 mM indole than by 1 mM indole. Conversely, 53 genes were downregulated by indole, 51 of which were more strongly repressed by 4 mM indole than by 1 mM indole. These data suggested that expression of most indole-regulated genes is indole concentration dependent. In fact, promoter activity of *ramA* increased as indole concentration increased. Similarly, expression of SPI-1 genes decreased as indole concentration increased.

**Figure 5:**
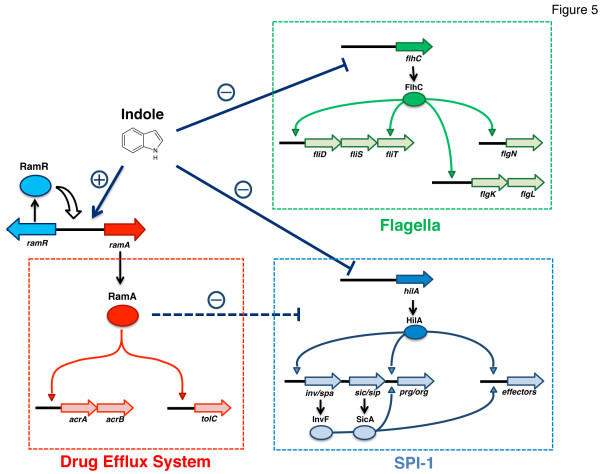
**Proposed regulatory network controlled by indole.** Indole induces the multidrug efflux system genes *acrAB* and *tolC* through the increased expression of *ramA*. Indole represses flagellar and SPI-1 genes in a *ram* locus-independent manner. However, the indole-mediated upregulation of *ramA* may be partially involved in decreased expression of SPI-1 genes.

Indole induced *ramA*, and this induction is probably responsible for induction of *acrB*, which encodes the multidrug efflux pump (Figure 5). Most of the genes upregulated by indole encode putative proteins. Several of the genes, such as STM1251, STM1472, STM1868A, STM3941, and STM4213, have yet to be named. Among these, the only gene identified in *E. coli* is STM4213, which encodes a putative phage tail sheath protein. If the function of these putative genes is clarified, then other phenotypes induced by indole in addition to multidrug resistance will also be understood.

In this study, we found that indole repressed various genes related to bacterial motility and virulence. Decreased motility and invasive activity of *Salmonella* were also phenotypically observed. Recent studies revealed the coordinate regulation of flagellar and SPI-1 genes by FliZ, encoded by a gene in the *fliA* operon [66,67]. It was demonstrated that FliZ posttranslationally controls HilD to positively regulate expression of *hilA*[67]. Our microarray data revealed reduced expression of *fliZ* when bacterial cells were treated with indole (*fliZ* expression was reduced to 0.47- and 0.038-fold of the level in untreated cells by 1 and 4 mM indole, respectively), and that expression of *hilA* was also repressed by indole. Indole may coordinately repress flagellar and SPI-1 genes via the regulatory network of FliZ. Because previous study indicated that both RamA and RamR are involved in the control of SPI-1 genes [47], we examined their effects on repression of SPI-1 genes and invasive activity of *Salmonella*. The results suggested that indole sup presses SPI-1 genes in a RamA/RamR-independent manner (Figure 5). Similarly, repression of *flhC* by indole was also RamA/RamR independent. These data are suggestive of the presence of another pathway for indole to repress flagellar and SPI-1 genes, whereas *acrAB*/*tolC* is induced by indole in a RamR/RamA-dependent manner. Bacterial adhesion to the Caco-2 cells, which is the primary step of the cell invasion process, was not addressed in our experiments. However, since flagella were repressed by indole, it cannot be excluded that the defective invasion partially resulted from a lesser adhesion to the cells, and not only to the repression of SPI-1 genes. It should also be noted that several of the indole-repressed genes are related to anaerobic respiration in addition to motility and SPI-1 genes. Because it is suggested that indole is a biological oxidant in bacteria [68], this oxidative effect may lead to repression of these genes.

In conclusion, we identified that indole induces expression of genes related to efflux-mediated multidrug resistance and represses expression of genes related to invasive activity and motility of *S. enterica* serovar Typhimurium. Reduction of invasive activity and motility of *Salmonella* by indole was phenotypically observed. Because *Salmonella* itself does not produce indole, our results suggest that indole could also be an important signaling molecule for inter-species communication to control drug resistance and virulence of *Salmonella* in addition to its role in intra-species communication in *E. coli*. Indeed, it was previously demonstrated that *E. coli*-conditioned medium induced the AcrAB pump in *Salmonella* through the RamA regulator [35]. The type of environment in which *Salmonella* experiences the effect of indole is not well understood. It is believed that *Salmonella* may be exposed to high indole concentrations in the intestine, in which several species of indole-producing bacteria exist. In fact, indole is found in human feces at comparable concentrations (~250–1100 μM) [29,30], and recent studies indicated the importance of indole in favorable inter-kingdom signaling interactions between the intestinal epithelial cells and commensal bacteria [69]. In addition to indole itself, indole derivatives such as skatole (3-methylindole) also occur naturally in feces after being produced from tryptophan in the mammalian digestive tract. Therefore, indole and skatole may additively affect gene expression in *Salmonella*. In fact, when we examined the effect of skatole by a β-galactosidase assay, it significantly stimulated the promoter activity of *ramA* (unpublished data). Thus, there is a possibility that these molecules enhance drug resistance of *Salmonella*, while simultaneously repressing their motility and pathogenicity in the intestinal tract. Interestingly, the effect of indole on the pathogenicity of *E. coli* is the opposite of that on *Salmonella*. In enterohemorrhagic *E. coli*, it was suggested that indole can activate expression of EspA and EspB as well as secretion and stimulate the ability of EHEC to form attaching and effacing lesions in human cells [38]. Thus, although indole secreted by *E. coli* enhances the virulence of *E. coli*, it reduces the virulence of *Salmonella*, probably to the advantage of *E. coli*. This finding suggests that the gastrointestinal flora may affect regulation of virulence traits in *Salmonella* via the signaling of indole.

## Competing interests

The authors declare that they have no competing interests*.*

## Authors’ contributions

Conceived and designed the experiments: EN, EG, SB, AC, KN. Performed the experiments: EN, EG, SB, SY, AW. Analyzed the data: EN, EG, SB, SY, AW, KO, TT, AY, AC, KN. Wrote the paper: EG, AC, KN. All authors read and approved the final manuscript.

## Funding

This research was supported in part by Grants-in-Aid from the Japan Society for the Promotion of Science and the Ministry of Education, Culture, Sports, Science and Technology of Japan; a grant from the Mishimakaiun Memorial Foundation; the Program for Promotion of Fundamental Studies in Health Sciences of the National Institute of Biomedical Innovation; and the Funding Program for Next Generation World-Leading Researchers. It was also partly supported by the French Région Centre (grant 2008 00036085) and partly by the European Union with the European Regional Development Fund (grant 1634–32245). The funders had no role in this study design, data collection and analysis, decision to publish, or preparation of the manuscript.
